# An Anatomical Study of At-Risk Nerves During Carpal Tunnel Release: Considerations for the Prevention of Iatrogenic Nerve Injury

**DOI:** 10.1177/22925503251379893

**Published:** 2025-09-25

**Authors:** Max J. Abercrombie, Kenneth Liu, Majid Alimohammadi

**Affiliations:** 1 Department of Cellular and Physiological Sciences, Faculty of Medicine, 8166 University of British Columbia, Vancouver, BC, Canada; 2 School of Kinesiology, Faculty of Education, University of British Columbia, Vancouver, BC, Canada

**Keywords:** carpal tunnel release, carpal tunnel syndrome, iatrogenic injury, nerve damage, palmar cutaneous branch of the median nerve, palmar cutaneous branch of the ulnar nerve, thenar motor branch, transverse carpal ligament, Libération du canal carpien, syndrome du canal carpien, lésion iatrogène, lésion nerveuse, branche cutanée palmaire du nerf médian, branche cutanée palmaire du nerf ulnaire, branche motrice de l’éminence thénar, ligament transverse du carpe

## Abstract

**Introduction:** Despite carpal tunnel release (CTR) being a common procedure in hand surgery, variation in the location of the nerves supplying the palm leads to a high risk of iatrogenic damage. Recommendations have been made for a surgical incision placement that would avoid such damage, yet injury persists in clinical practice. These studies infrequently consider the safety of multiple at-risk nerves when making their recommendation, often optimizing the safety of one and subsequently jeopardizing another's. **Methods:** Sixty-one dissections were performed on formalin preserved cadavers to define a safe zone in the palm and recommend an incision placement for CTR. Detailed measurements examining the anatomy of the palmar cutaneous branch of the median nerve (PCBMN), the palmar cutaneous branch of the ulnar nerve (PCBUN), and the thenar motor branch (TMB) were taken relative the scaphoid tubercle, pisiform, or the A line. **Results:** The PCBMN was located 3.3 ± 4.1 mm ulnar to the scaphoid tubercle and 8.7 ± 3.9 mm radial to the A line. The PCBUN was located the 6.5 ± 2.4 mm radial and 6.6 ± 3.7 mm ulnar from the pisiform and A line respectively. The TMB was found 8.0 ± 3.3 mm from the A line and was classified as 56% extraligamentous, 31% subligamentous, and 13% transligamentous. **Conclusion:** We conclude that an area approximately 6 mm ulnar and 7 mm radial from the A line is the safe zone for CTR and recommend an incision placement in line with the radial aspect of the fourth digit. This knowledge may aid surgeons performing CTR and help reduce iatrogenic damage.

## Résumé

**Introduction :** Bien que la libération du canal carpien (LCC) soit une procédure courante dans la chirurgie de la main, la variation de l’emplacement des nerfs innervant la paume entraîne un risque élevé de dommages iatrogéniques. Des recommandations ont été faites sur l’emplacement de l’incision chirurgicale dans le but d’éviter de telles lésions, mais des blessures persistent néanmoins en pratique clinique. Rarement, ces études tiennent compte de la sécurité de multiples nerfs en danger lorsqu’elles formulent leurs recommandations, se concentrant souvent sur l’optimisation de la sécurité d’un nerf et en mettant, par conséquent, un autre en danger. **Méthodes :** Soixante et une dissections ont été effectuées sur des cadavres préservés dans le formol pour définir une zone sécuritaire dans la paume et recommander l’emplacement de l’incision pour la LCC. Des mesures détaillées de l’anatomie de la branche cutanée palmaire du nerf médian (PCBMN), de la branche cutanée palmaire du nerf ulnaire (PCBUN) et de la branche motrice de l’éminence thénar (TMB) ont été prises par rapport au tubercule scaphoïdien, au pisiforme ou à la ligne A. **Résultats :** Le PCBMN était situé à 3,3 ± 4,1 mm en situation ulnaire par rapport au tubercule scaphoïde et à 8,7 ± 3,9 mm en situation radiale par rapport à la ligne A. Le PCBUN était situé à 6,5 ± 2,4 mm en situation radiale et à 6,6 ± 3,7 mm en situation ulnaire, respectivement, du pisiforme et de la ligne A. La TMB a été trouvée à 7,9 ± 3,3 mm de la ligne A et a été classée extraligamentaire dans 56% des cas, sous-ligamentaire dans 32% des cas et transligamentaire dans 13% des cas. **Conclusion :** Nous en concluons qu’une zone située à approximativement 6 mm en ulnaire et 7 mm en radial de la ligne A constitue la zone sécuritaire pour la LCC et nous recommandons de placer l’incision dans l’alignement du bord radial du quatrième doigt. Cette information peut aider les chirurgiens dans la réalisation d’une LCC et contribuer à diminuer les lésions iatrogènes.

## Introduction

Carpal tunnel syndrome results from compression of the median nerve and is the most common peripheral compressive neuropathy.^1,2^ Surgical treatment through carpal tunnel release (CTR) is one of the most frequent procedures in hand surgery and involves transecting the transverse carpal ligament (TCL) to release pressure on the median nerve.^1,2^ However, research has shown that there is wide variation in the course of the palmar cutaneous branch of the median nerve (PCBMN),^3–9^ the palmar cutaneous branch of the ulnar nerve (PCBUN),^9–14^ and the thenar motor branch (TMB) of the median nerve,^13,15–18^ which needs to be considered during CTR.

Due to the high degree of variability of these nerves and their location within the surgical area, they are at risk of being damaged with CTR.^1^ Complications of CTR involving these structures leads to various morbidities related to the damaged nerves such as neuromas, paresthesia, and loss of function.^1,2^ The prevalence of injury to the PCBMN and TMB are reported to be 0.03% and 0.01% respectively.^1^ Although specific data for the prevalence of iatrogenic injury to the PCBUN has not been reported, ulnar nerve damage occurs in 0.03% of cases.^2^ As CTR is a common procedure with a rate of 10 patients per 10 000 Canadians,^19^ the incidence of nerve injury represents a significant number of patients. Despite a growing body of evidence, injury to these nerves persists in clinical practice and further research into their complete course is warranted.

Numerous recommendations have been made as to the placement of a surgical incision that would minimize damage to these structures.^3,6,9,13,14,20^ However, due to the use of inconsistent landmarks and varying measurements, results remain contradictory and further data collection is required. Additionally, studies infrequently consider the safety of all three of these nerves when making their recommendation, often optimizing the safety of one and subsequently jeopardizing another's. A comprehensive understanding of the course and variance of these nerves in conjunction with one another is critical for avoiding iatrogenic damage from CTR.

This study aimed to collect comprehensive data on the position of the PCBMN, TMB, and PCBUN with reference to consistent anatomical landmarks relevant for CTR. With this knowledge, we aimed to suggest a safe zone in the palm and provide a recommendation for the placement of the incision to aid surgeons performing CTR. Utilizing statistical analysis, we aimed to examine the variation of measurements between TMB classifications, quantify the symmetry of TMB classification between sides, record differences between sides within a subject, and document sex differences.

## Materials and Methods

### Cadavers

Sixty-one (30 left, 31 right) formalin preserved upper limbs were dissected and analyzed from 31 cadavers (13 female, 18 male). Dissections were bilateral in 30 specimens and unilateral in a single specimen. All available data was collected depending on the condition of each forearm or palm (24 intact limbs, 26 pre-dissected limbs, 11 palm only). Cadavers were provided through the institution's Gross Anatomy Lab. The study protocol conformed to the ethical guidelines of the 1975 Declaration of Helsinki and was approved by the department's oversight committee for this research. Informed consent was obtained for the use of anatomic donations.

### Data Collection

**Figure 1.**
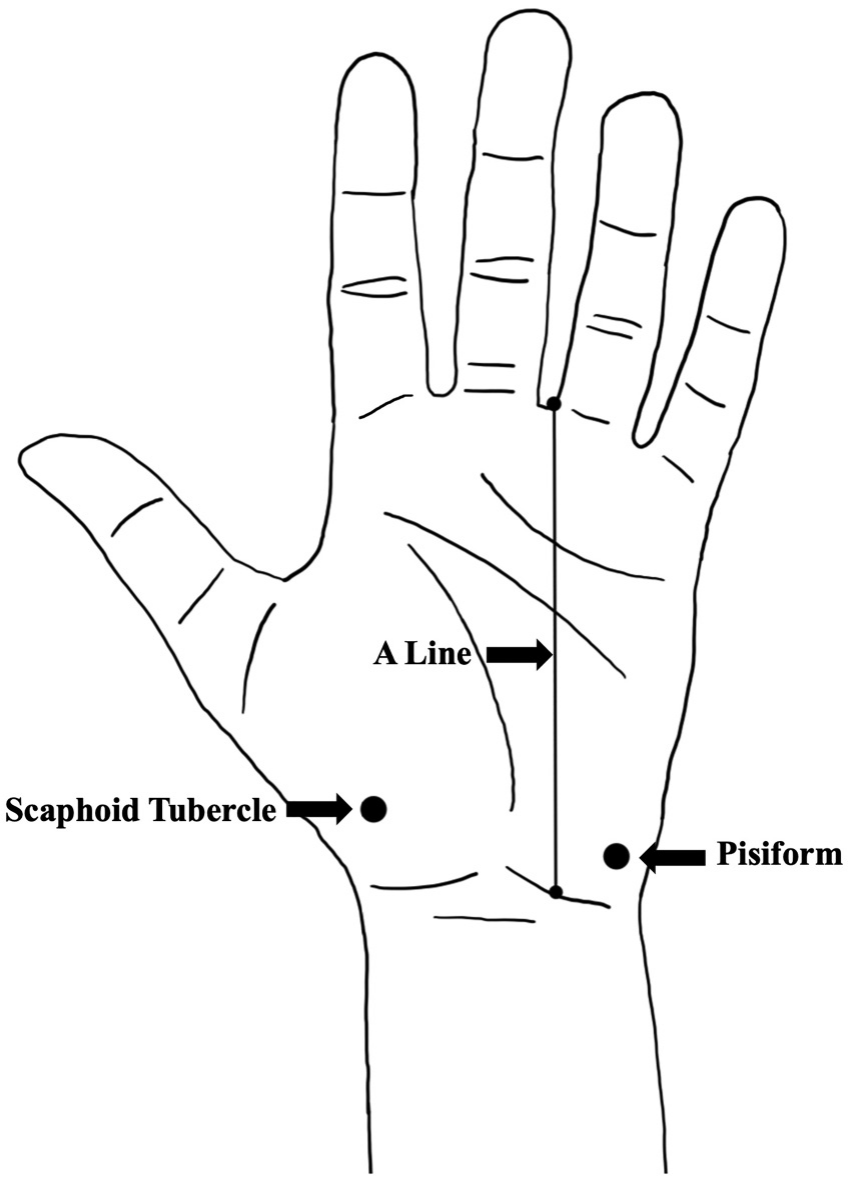
Boney landmarks and reference points used for measurements.

A consistent method was utilized for each dissection with the starting point depending on the condition of the limb. A longitudinal, cutaneous incision was made in line with the third web space connecting two horizontal incisions in the proximal forearm and distal palm. The skin was reflected, and the scaphoid tubercle (ST) and pisiform were palpated and marked to be used as anatomical reference points. A longitudinal line was established from the radial edge of the fourth digit hereby referred to as the A line (Figure 1). All measurements were taken using a Vernier caliper.

Using blunt dissection, the median nerve was identified in the distal forearm between the tendons of the flexor carpi radialis and palmaris longus when present. The PCBMN was then identified and the point of radial deviation from the median nerve was measured to the ST. The PCBMN was dissected back to its true, intraneural origin where the distance was measured to the ST. Finally, the PCBMN and any branches were dissected to the level of the ST where the horizontal distance from the most ulnar border or branch was measured to the ST and A line. The PCBMN was used as the proximal radial margin for the safe zone.

The ulnar nerve was identified in the distal forearm radial and deep to the flexor carpi ulnaris on the surface of the flexor digitorum profundus. The PCBUN was identified and dissected back to its true, intraneural origin where the distance was measured to the pisiform. In the distal forearm, the point of radial deviation off the ulnar artery was observed if present and measured to the pisiform. Next, the PCBUN and any branches were dissected to the level of the pisiform where horizontal measurements from the most radial border or branch were taken to the pisiform and the A line. The PCBUN was used as the ulnar margin for the safe zone.

To observe the median nerve within the palm, the palmar aponeurosis and TCL were cut and reflected. Using blunt dissection, the TMB of the median nerve was identified and classified. At both the origin of the TMB and its insertion into the thenar eminence (TE), the distance was measured distal to the ST and radial to the A line. The TMB was used as the distal radial margin for the safe zone.

### Data Analysis

Following data collection, mean values and standard deviations were calculated for all measurements. All data is presented in means ± standard deviation. Qualitative observations were reviewed for consistent and outlier information to help inform the presentation of the measurements. Graphs were created in Excel version 16.96 (Microsoft Corporation).

### Statistical Analysis

Statistical analysis was conducted using IBM SPSS Statistics version 29 (IBM Corporation) and R statistical software version 2025.05.1 + 513 (R Foundation for Statistical Computing) with statistical significance set at *P* ≤ .05. Chi-squared test for independence was utilized to assess the relation of sex and side to TMB classification. Paired-samples *T* tests were utilized to examine each measurement between left and right sides. Independent samples *T* tests were utilized to examine the effect of sex as well as limb condition on each measurement recorded. Additionally, an independent samples *T* test was used to examine the distance to the A line at the TE in extraligamentous versus subligamentous TMB classifications. One-way analysis of variance (ANOVA) tests were utilized to examine the variance in measurements between TMB classifications. Tukey HSD testing was then run to assess the relationship between TMB classifications.

## Results

### Palmar Cutaneous Branch of the Median Nerve

**Figure 2.**
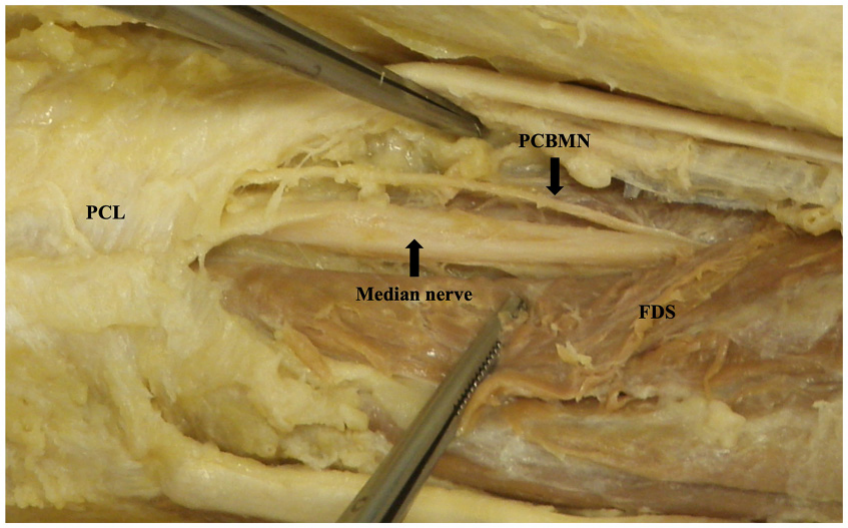
Palmar cutaneous branch of the median nerve dissected back to origin. Image is of the right limb positioned distal (left) to proximal (right).

Abbreviations: FDS, flexor digitorum superficialis; PCBMN, palmar cutaneous branch of the median nerve; PCL, palmar carpal ligament.

**Figure 3.**
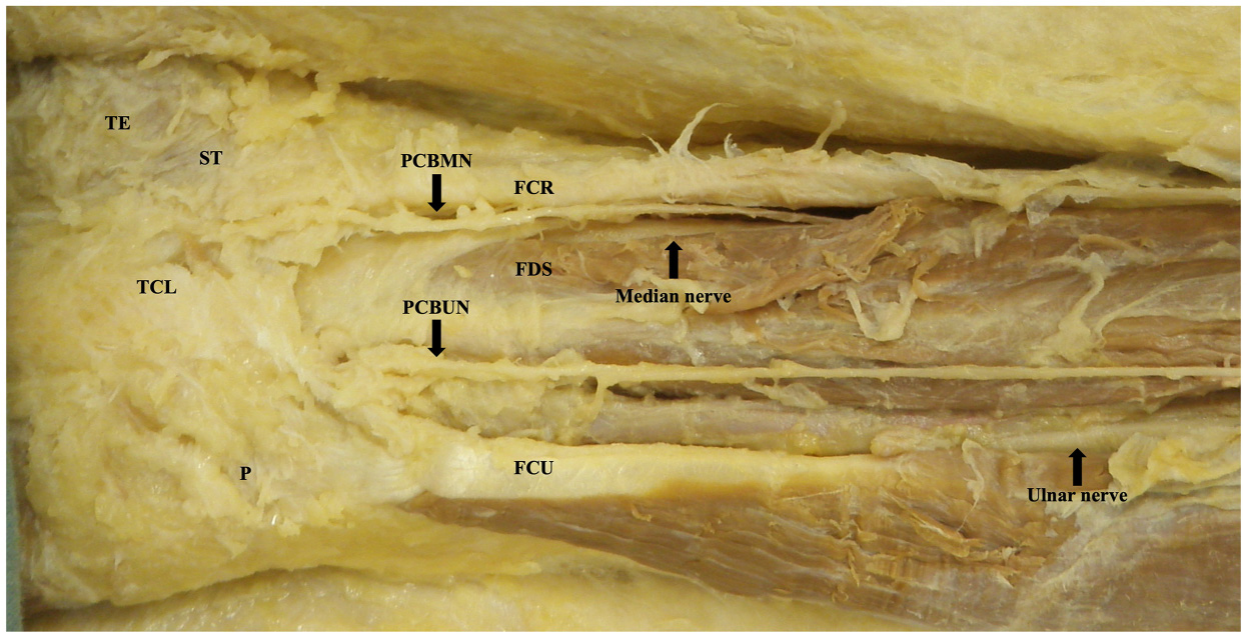
Dissected course of palmar cutaneous branch of the median and ulnar nerves in the wrist and palm. Image is of the right limb positioned distal (left) to proximal (right).

Abbreviations: TE, thenar eminence; TCL, transverse carpal ligament; ST, scaphoid tubercle; P, pisiform; FCR, flexor carpi radialis; FDS, flexor digitorum superficialis; FCU, flexor carpi ulnaris; PCBMN, palmar cutaneous branch of the median nerve; PCBUN, palmar cutaneous branch of the ulnar nerve.

**Figure 4.**
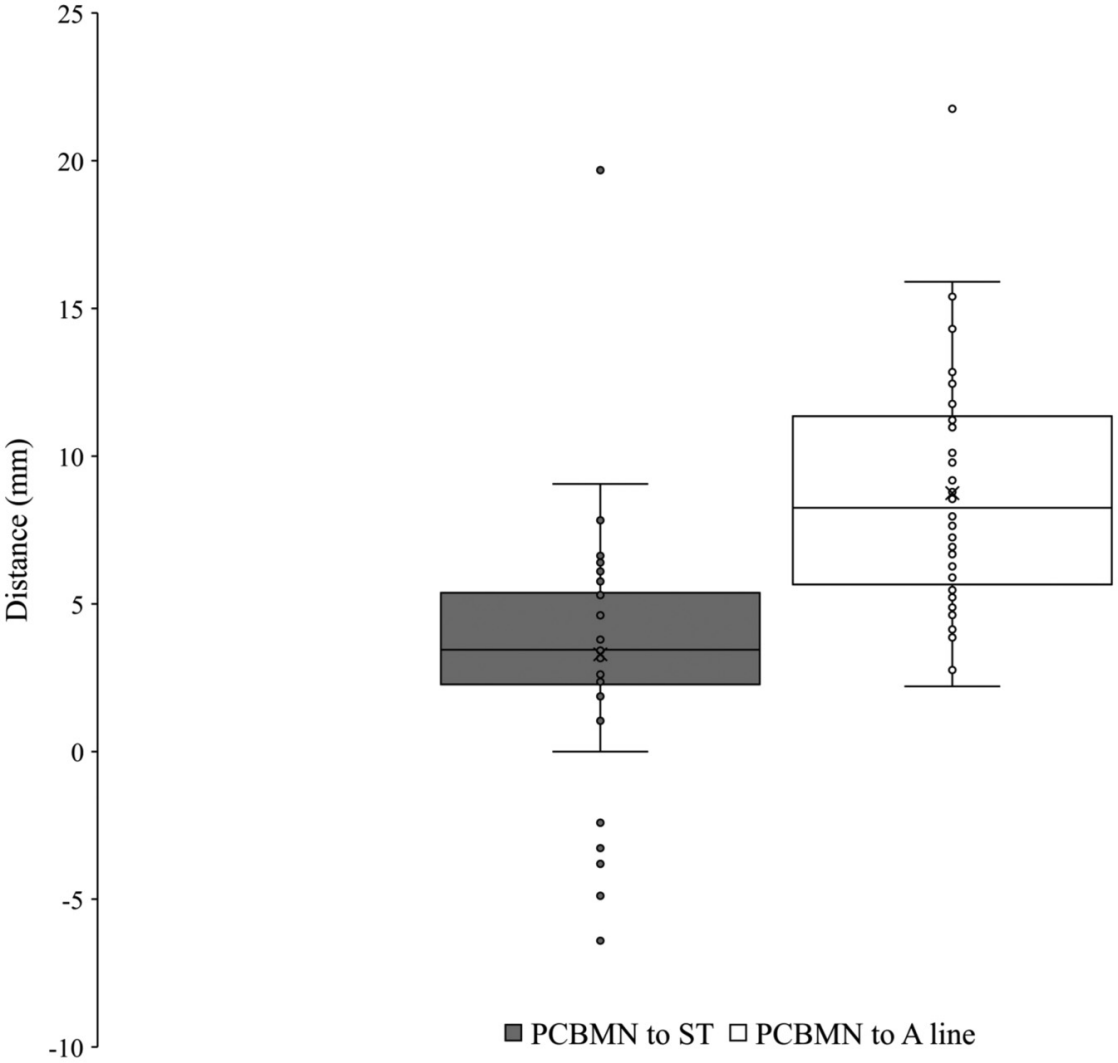
Variation of the palmar cutaneous branch of the median nerve in the wrist.

Abbreviations: PCBMN, palmar cutaneous branch of the median nerve; ST, scaphoid tubercle.

The median nerve was found ulnar to the tendon of flexor carpi radialis and radial to the tendon of flexor digitorum superficialis and palmaris longus. Present in 100% (*n* = 50) of dissections, the PCBMN originated from the radial aspect of the median nerve 75.7 ± 16.3 mm from the ST (Figure 2). The PCBMN then travelled in the sheath of the median nerve before radially deviating off the main trunk 37.6 ± 10.1 mm from the ST. Following radial deviation, the PCBMN travelled below the proximal portion of the antebrachial fascia and entered the palmar carpal ligament forming its own tunnel. The PCBMN consistently entered the palm radial to the flexor digitorum superficialis and ulnar to the flexor carpi radialis. The PCBMN branched proximal to the ST in 90% (*n* = 45) of dissections. At the level of the ST, the PCBMN was observed to be an average of 3.3 ± 4.1 mm ulnar to the ST and 8.7 ± 3.9 mm radial to the A line (Figure 3). Figure 4 graphically represents the variation of the PCBMN in the proximal palm. In a single dissection, the PCBMN was observed to have crossed the A line and would have been transected by an incision typically used for CTR. All PCBMN measurements are presented in Table 1.

**Table 1.** Palmar Cutaneous Branch of the Median Nerve Data.

Measurement	*N*	Average (mm)	Standard deviation (mm)
Origin of PCBMN to ST	50	75.7	16.3
Point of radial deviation to ST	50	37.6	10.1
Distance to ST at level of ST	46	3.3	4.1
Distance to A line at level of ST	46	8.7	3.9

Abbreviations: ST, scaphoid tubercle; PCBMN, palmar cutaneous branch of the median nerve.

The point of radial deviation to the ST was found to be statistically significantly greater in right compared to left sides (Mean Left–Right difference −5.5 ± 12.0 mm, 95% CI [−11.0, −0.02], *P* < .05). No other measurement was found to be statistically significant (*P* > .05) between sex, side, or limb condition.

### Palmar Cutaneous Branch of the Ulnar Nerve

**Figure 5.**
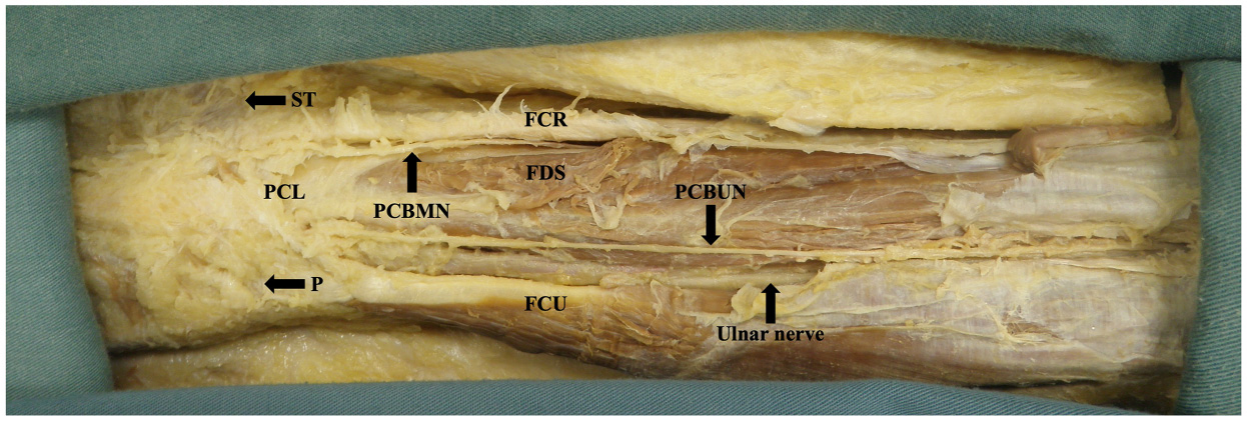
Dissected course of the palmar cutaneous branch of the median and ulnar nerves in the forearm and proximal palm. Image is of the right limb positioned distal (left) to proximal (right).

Abbreviations: ST, scaphoid tubercle; PCL, palmar carpal ligament; P, pisiform; PCBMN, palmar cutaneous branch of the median nerve; FCR, flexor carpi radialis; FDS, flexor digitorum superficialis; FCU, flexor carpi ulnaris; PCBUN, palmar cutaneous branch of the ulnar nerve.

**Figure 6.**
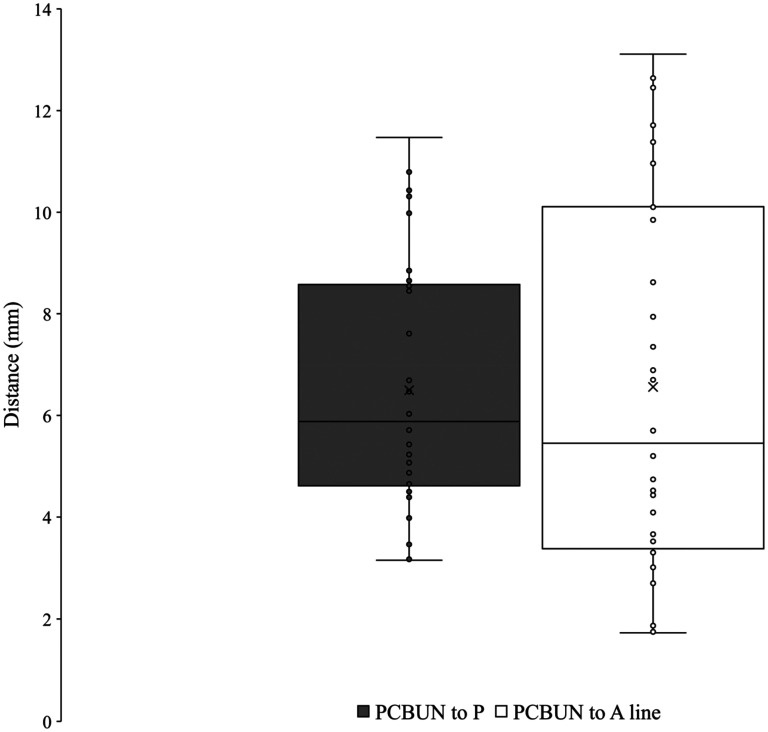
Variation of the palmar cutaneous branch of the ulnar nerve in the wrist.

Abbreviations: PCBUN, palmar cutaneous branch of the ulnar nerve; P, pisiform.

The ulnar nerve was found with the ulnar artery radial and deep to the flexor carpi ulnaris on the anterior surface of the flexor digitorum profundus. The PCBUN was present in 89% of dissections (*n* = 34). The origin of the PCBUN was found 150.4 ± 23.0 mm to the pisiform and was consistently located in the proximal forearm where the ulnar artery and ulnar nerve converge to lie subjacent to one another (Figure 5). The PCBUN was observed to deviate from the ulnar nerve at or briefly after its origin and travel on the superficial surface of the ulnar artery. In the distal forearm, the PCBUN was observed in 22 cases to radially deviate off the ulnar artery an average of 35.7 ± 16.1 mm from the pisiform. Entering the palm, the PCBUN travelled ulnar to the tendon of palmaris longus and radial to the tendon of flexor carpi ulnaris in 100% of dissections. The PCBUN branched proximal to the pisiform in 53% (*n* = 18) of dissections. At the level of the pisiform, the PCBUN was observed to be an average of 6.5 ± 2.4 mm radial and 6.6 ± 3.7 mm ulnar from the pisiform and A line, respectively (Figure 3). Figure 6 graphically represents the variation of the PCBUN in the proximal palm. Nerves that radially deviated (*n* = 22) were on average 1.4 mm closer to the A line compared to those that did not (*n* = 12). All PCBUN measurements are presented in Table 2.

**Table 2.** Palmar Cutaneous Branch of the Ulnar Nerve Data.

Measurement	*N*	Average (mm)	Standard deviation (mm)
Origin of PCBUN to P	34	150.4	23.0
Point of radial deviation to P	22	35.7	16.1
Distance to P at level of P	30	6.5	2.4
Distance to A line at level of P	30	6.6	3.7

Abbreviations: P, pisiform; PCBUN, palmar cutaneous branch of the ulnar nerve.

The distance to the pisiform from the origin of the PCBUN was found to be statistically significantly different between sexes (Mean Female–Male difference −27.6 ± 6.4 mm, 95% CI [−40.5, −14.6], *P* < .001) with a greater distance recorded in males. No other measurement was found to be statically significant (*P* > .05) between sex, side, or limb condition.

### Thenar Motor Branch

**Figure 7.**
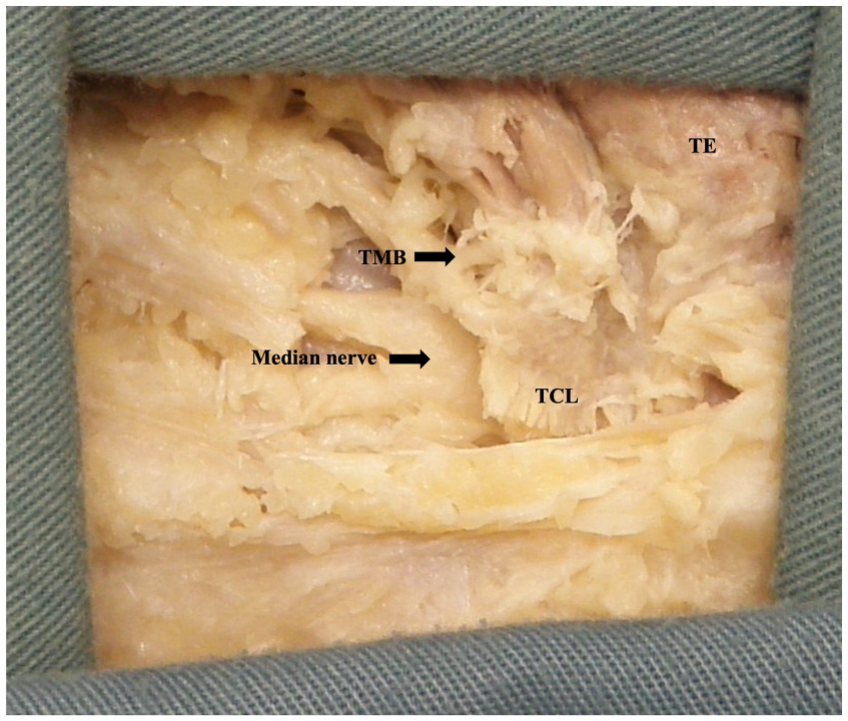
Extraligamentous thenar motor branch. Image is of the left limb in prone positioned distal (left) to proximal (right).

Abbreviations: TMB, thenar motor branch; TCL, transverse carpal ligament; TE, thenar eminence.

**Figure 8.**
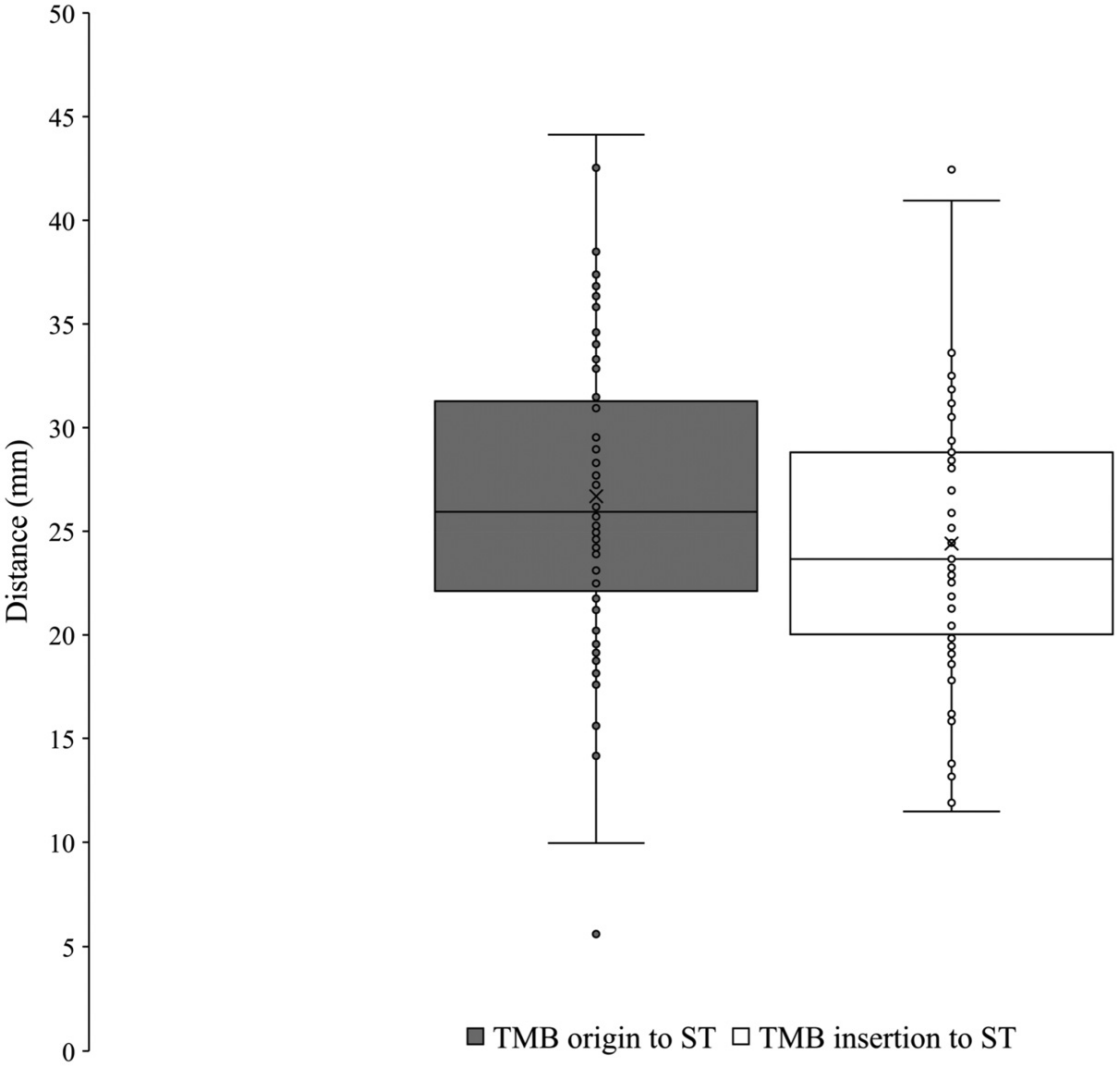
Variation of the thenar motor branch measured to scaphoid tubercle.

Abbreviations: TMB, thenar motor branch; ST, scaphoid tubercle.

**Figure 9.**
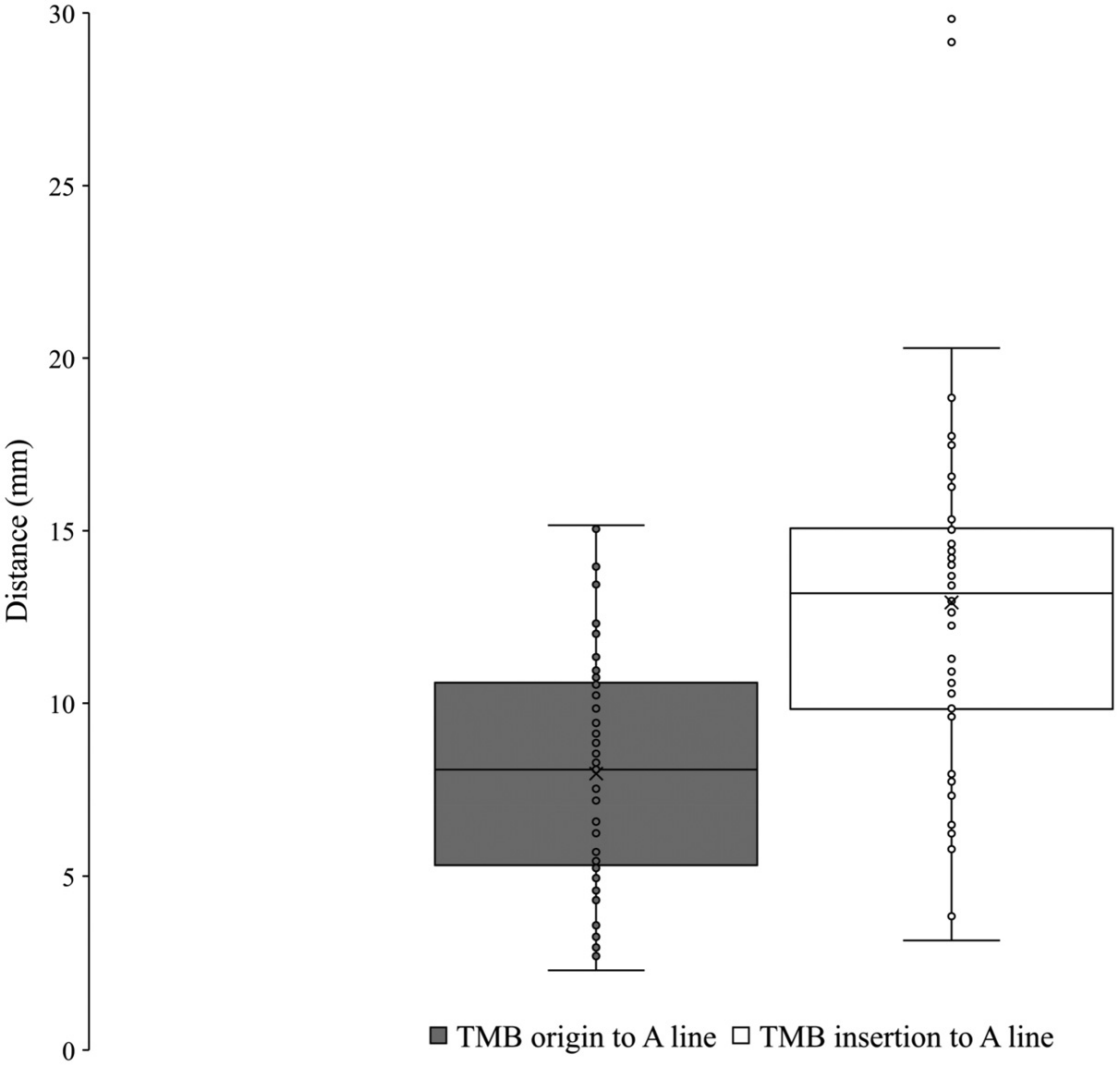
Variation of the thenar motor branch measured to A line. TMB, thenar motor branch.

The TMB was observed in all palms dissected (*n* = 61). Using Lanz group I classification, the prevalence was observed at 56% extraligamentous, 31% subligamentous, and 13% transligamentous (Figure 7). At the origin from the main trunk of the median nerve, the TMB was observed to be an average of 26.7 ± 7.4 mm and 8.0 ± 3.3 mm from the ST and A line respectively. At the insertion to the TE, the TMB was observed to be an average of 24.4 ± 6.6 mm and 12.9 ± 5.2 mm from the ST and A line, respectively. Figures 8 and 9 graphically represent the variation of the TMB measured to the ST and A line, respectively. Measurements are presented for all three classifications in Tables 3 to 5.

**Table 3.** Extraligamentous Thenar Motor Branch Data.

Measurement	*N*	Average (mm)	Standard deviation (mm)
Distance to ST at origin	34	28.8	6.8
Distance to A line at origin	34	8.1	3.4
Distance to ST at TE	32	24.2	6.8
Distance to A line at TE	32	12.3	5.1

Abbreviations: ST, scaphoid tubercle; TE, thenar eminence.

**Table 4.** Subligamentous Thenar Motor Branch Data.

Measurement	*N*	Average (mm)	Standard deviation (mm)
Distance to ST at origin	19	25.3	5.3
Distance to A line at origin	19	7.6	3.4
Distance to ST at TE	15	24.9	6.1
Distance to A line at TE	15	14.3	5.3

Abbreviations: ST, scaphoid tubercle; TE, thenar eminence.

**Table 5.** Transligamentous Thenar Motor Branch Data.

Measurement	*N*	Average (mm)	Standard deviation (mm)
Distance to ST at origin	8	21.0	11.2
Distance to A line at origin	8	8.0	3.0
Distance to ST at TE	5	24.0	8.1

Abbreviations: ST, scaphoid tubercle; TE, thenar eminence.

### Thenar Motor Branch Statistics

There was no statistically significant association between classification and sex (*P* > .05) or side (*P* > .05) nor between limb condition (*P* > .05). The distance between the origin of the TMB and the ST was found to be statistically significantly different (Mean Extraligamentous–Transligamentous difference 7.7 ± 2.8 mm, 95% CI [1.1, 14.4], *P* < .05) between extraligamentous and transligamentous classifications. No other measurement presented statistical significance (*P* > .05) between TMB classifications. The bilateral symmetry of TMB classification within subjects was found to be 47% (*n* = 14).

## Discussion

### Carpal Tunnel Release Incision

The primary aim of this study was to identify a safe zone in the palm and recommend an incision placement for CTR. Utilizing the PCBUN ulnarly and the PCBMN as well as TMB radially, data was collected to establish the margins of the safe zone. From these findings, we suggest that an area approximately 6 mm ulnar and 7 mm radial from the A line is the safe zone for CTR. With this knowledge, we recommend that the incision for CTR be placed in line with the radial aspect of the fourth digit to minimize the risk of iatrogenic damage to these nerves.

### Palmar Cutaneous Branch of the Median Nerve

Measurements of the PCBMN have wide variation in the literature due in part to the use of inconsistent landmarks. Many studies have utilized the distal wrist crease (DWC) as a reference point for measurements as this cutaneous landmark is easily visualized and positioned above the proximal border of the TCL.^6,7,13,20–23^ However, variation in the position of the DWC has been demonstrated^8^ which may introduce unreliability into findings and likely contributes to inconsistent measurements between studies. For example, studies by Hobbs et al^21^ and Cheung et al^20^ both utilized the DWC yet recorded average measurements of 8.4 cm and 3.2 cm to the origin respectively. For this reason, consistent, boney landmarks (ie, ST or pisiform) were utilized in this study to minimize any variance due to reference points. In the current study, the origin of the PCBMN was observed to be 75.7 ± 16.3 mm from the ST. This finding coincides with Richards et al^24^ who reported an average distance of 76.04 mm from the origin to the ST. The use of consistent, palpable, boney landmarks allows for more accurate comparisons of results between studies and can aid surgeons in reliably mapping the incision placement topographically.

At the level of the ST in the palm, the PCBMN is at risk of being transected during CTR. This study recorded the position of the PCBMN an average of 3.3 ± 4.1 mm ulnar to the ST and 8.7 ± 3.9 mm radial to the A line. Comparing these findings to the current body of evidence is important in justifying the measurement's clinical relevance for CTR. However, the comparison of data between studies is made challenging by the use of varying reference points. At the level of the DWC, a study by Watchmaker et al^7^ observed the PCBMN to be an average of 2 mm radial to the thenar crest. Additionally, Xu et al^9^ reported the PCBMN to be an average of 6.10 mm from a line down the middle of the third digit at the DWC. As these studies all measured at the level of the DWC and to varying longitudinal reference points, results are difficult to compare to the current study and further emphasizes the demand for consistent methodology.

### Palmar Cutaneous Branch of the Ulnar Nerve

Due to the scarcity of information surrounding the PCBUN, the findings of this study contribute significantly to the current body of evidence. Absent in 4 of 38 cadavers, the PCBUN was present in 89% (*n* = 34) of limbs dissected. This finding is supported by other studies that have found the PCBUN not to be a constant structure with a prevalence ranging from 67% to 90%.^9,13,14^ The origin of the PCBUN was found in the proximal forearm an average of 150.4 ± 23.0 mm to the pisiform. This finding supports studies reporting a more proximal origin, such as Engber and Gmeiner^10^ and Xu et al,^9^ who observed an origin 14 cm from the pisiform and 10.12 cm from the DWC, respectively. Previously recorded variations of the nerve including a more distal origin have been reported,^6,11,13^ but were not observed in the current study.

With many studies examining the position of the PCBMN in the palm and suggesting a more ulnar incision for CTR,^3,20^ an understanding of the position of the PCBUN in the palm is of great clinical significance. However, limited quantitative data exists for such an observation in the current literature. At the level of the DWC, Xu et al^9^ reported the PCBUN to be an average of 4.7 mm from a longitudinal line of the third finger. In the current study, the PCBUN was found to be an average of 6.5 ± 2.4 mm radial and 6.6 ± 3.7 mm ulnar from the pisiform and A line, respectively. This finding suggests that an incision placed on the ulnar aspect of the fourth digit may risk transection of the PCBUN.

### Thenar Motor Branch

The findings of this study contribute to the large body of evidence examining the TMB and its course using Lanz^15^ classification. The observed prevalence of TMB classifications in the current study are supported by a recent meta-analysis by Henry et al^18^ who reported a pooled prevalence from 3918 hands at 75.2% extraligamentous, 13.5% subligamentous, and 11.3% transligamentous.

The TMB was utilized as the distal reference for the radial margin of the safe zone. At the origin from the median nerve and insertion to the TE, the TMB was found to be an average of 8.0 ± 3.3 mm and 12.9 ± 5.2 mm from the A line, respectively. Studies by Ozcanli et al,^13^ Eskandari et al,^17^ and Wilhelmi et al^16^ utilized a similar longitudinal reference point to the current study reporting the origin of the TMB to be 7.3 mm, 12.6 mm, and 8.6 mm radial, respectively.

### Limitations

The cadavers provided for this study were additionally used for teaching purposes and the condition of the limb varied in pre-dissected (*n* = 26) cadavers. As some nerves were not preserved during previous dissections, data collection on all the structures of interest was not possible in every cadaver. This limited the number of cadavers where a whole data set could be collected, yet the maximal amount of data was extracted from each limb.

Previous descriptions of the PCBMN and PCBUN reporting more distal observations have observed branching within the palm that may course toward the surgical area.^6–9,13,14,21^ In the current study, measurements of the PCBMN and PCBUN were taken as far distally as the ST and pisiform, respectively. As such, the current study does not account for any distal branching which may occur in some variations.

## Conclusion

Through data collected on the PCBMN, TMB, and PCBUN from 61 dissections, this study has defined a safe zone for CTR and contributed a large data set to the field of study. We conclude that an area approximately 6 mm ulnar and 7 mm radial from a longitudinal line on the radial aspect of the fourth digit is the safe zone for CTR. Therefore, this study recommends an incision in line with the radial aspect of the fourth digit to minimize the risk of iatrogenic damage during CTR. This knowledge can aid surgeons performing CTR and increase the safety of the procedure.

## Supplemental Material

**Figure:** Video 1.
